# O23 A series of catastrophic events

**DOI:** 10.1093/rap/rkab067.022

**Published:** 2021-10-19

**Authors:** Samir Patel, Spencer Ellis, David D'Cruz

**Affiliations:** 1 Guy's and St. Thomas' hospital, London, United Kingdom; 2 Lister hospital, Stevenage, United Kingdom

## Abstract

**Case report - Introduction:**

Catastrophic antiphospholipid syndrome (CAPS) is a rare, life-threatening disease occurring in up to 1% of antiphospholipid syndrome (APS) cases. It was first defined in 1992 and remains a difficult to treat entity with a mortality rate of 37%. We describe a patient with systemic lupus erythematosus (SLE) and CAPS presenting with simultaneous multi-organ injuries who was successfully managed with ‘triple’ therapy including cyclophosphamide.

**Case report - Case description:**

A 42-year-old female presented to her local hospital with chest pain and worsening vision. She had a background of SLE, triple antibody-positive APS (previous DVT, pregnancy loss and strokes), hypertension, a metallic mitral valve, a previous myocardial infarction and pre-existing visual impairment due to a prior intra-cerebral bleed related to anticoagulation. Examination revealed a faint malar rash, cortical blindness and long tract neurological signs. Her ECG showed ischaemic changes and the admission troponin was significantly raised (3773ng/L). An echocardiogram showed new left ventricular dysfunction and a subsequent cardiac MRI was in keeping with coronary artery disease. Investigations showed an acute kidney injury, newly deranged liver function tests and a raised INR (>11, with no bleeding). Complement was normal with a low dsDNA titre. Urinalysis revealed proteinuria and a protein creatinine ratio measured 176mg/mmol. MRI diffusion weighted brain imaging showed acute bilateral occipital and left fronto-parietal infarcts. She had symptoms of a lupus flare with arthralgia and a butterfly facial rash.

COVID-19 PCR tests were negative and she had not been recently vaccinated. She was diagnosed with CAPS and transferred to St Thomas’ hospital intensive care.

On arrival, she received 1mg intravenous vitamin K followed by triple therapy for CAPS: an unfractionated heparin infusion, oral prednisolone 40mg daily, 5 days of plasma exchange and, given her background of SLE, she was treated with intravenous cyclophosphamide (according to the EUROLUPUS regimen). Intravenous methylprednisolone was avoided due to a previous hypertensive encephalopathy reaction.

She responded rapidly. Her troponin fell from a peak of 5054 to 294ng/L, her creatinine settled at a new baseline (232umol/L) and her liver function normalised. She was switched back to warfarin due to her metallic valve and started on aspirin for cardiovascular secondary prevention. She required physical and occupational therapy due to her strokes but recovered well.

**Case report - Discussion:**

According to the 2003 criteria, CAPS can be classified as definite when there is evidence of: ≥3 organs involved, development of manifestations simultaneously or within a week, confirmation by imaging and/or histopathology of small vessel occlusion and positive antiphospholipid antibodies. Probable CAPS is when 3 out of the 4 criteria are present.

In this case, three organs were confirmed to be involved with imaging showing cerebral and cardiac ischaemia. Her creatinine rose from a base of 190 to 289umol/L coupled with a high protein creatinine ratio confirming renal involvement. A Budd-Chiari syndrome was also suspected due to deranged liver function tests and INR, though imaging performed after therapy did not confirm this. A biopsy of any of these four organs was not feasible given the severity of her presentation and coagulopathy.

There are no randomised controlled trials but data from the CAPS registry guides treatment and management follows a logical approach: anticoagulation to treat thrombosis, glucocorticoids for inflammation and plasma exchange (or IVIG) to remove the circulating autoantibodies. Triple therapy was associated with a reduced mortality compared to no treatment (28.6% versus 75%, respectively).

Following analyses from the CAPS registry we also chose to treat with cyclophosphamide, which is associated with improved survival in patients with SLE. This decision was based on the clinical features of an SLE flare as opposed to serological grounds. There have been reports of rituximab and eculizumab being used successfully in CAPS, though generally as a last resort. As complement activation is seen in animal models of antiphospholipid syndrome thrombosis and rituximab is often used in refractory SLE, they may prove to be promising agents for refractory CAPS.

**Case report - Key learning points:**

1. Prompt recognition and early treatment is vital in managing CAPS
2. Triple therapy with anticoagulation, glucocorticoids and plasma exchange / IVIG is associated with better survival in CAPS
3. Cyclophosphamide is associated with better survival in patients with CAPS and concomitant SLE

